# Echocardiographic Prognosis Relevance of Attenuated Right Heart Remodeling in Idiopathic Pulmonary Arterial Hypertension

**DOI:** 10.3389/fcvm.2021.650848

**Published:** 2021-05-07

**Authors:** Qin-Hua Zhao, Su-Gang Gong, Rong Jiang, Chao Li, Ge-Fei Chen, Ci-Jun Luo, Hong-Ling Qiu, Jin-Ming Liu, Lan Wang, Rui Zhang

**Affiliations:** ^1^Department of Pulmonary Circulation, Shanghai Pulmonary Hospital, Tongji University School of Medicine, Shanghai, China; ^2^Tongji University School of Medicine, Shanghai, China; ^3^Department of Biosciences and Nutrition, Karolinska Institutet, Stockholm, Sweden

**Keywords:** pulmonary arterial hypertension, right heart remodeling, echocardiography, biomarkers, prognosis

## Abstract

**Background:** Right ventricular (RV) function is a great determination of the fate in patients with pulmonary arterial hypertension (PAH). Monitoring RV structure back to normal or improvement should be useful for evaluation of RV function. The aims of this study were to assess the prognostic relevance of changed right heart (RH) dimensions by echocardiography and attenuated RH remodeling (ARHR) in idiopathic PAH (IPAH).

**Methods:** We retrospectively analyzed 232 consecutive adult IPAH patients at baseline assessment and included RH catheterization and echocardiography. ARHR at the mean 20 ± 12 months' follow-up was defined by a decreased right atrium area, RV mid-diameter, and left ventricular end-diastolic eccentricity index. The follow-up end point was all-cause mortality.

**Results:** At mean 20 ± 12 months' follow-up, 33 of 232 patients (14.2%) presented with ARHR. The remaining 199 surviving patients were monitored for another 25 ± 20 months. At the end of follow-up, the survival rates at 1, 3, and 5 years were 89, 89, and 68% in patients with ARHR, respectively, and 84, 65 and 41% in patients without ARHR (log-rank *p* = 0.01). ARHR was an independent prognostic factor for mortality. Besides, ARHR was available to further stratify patients' risk assessment through the French PAH non-invasive-risk criteria.

**Conclusions:** Echocardiographic ARHR is an independent determinant of prognosis in IPAH at long-term follow-up. ARHR might be a useful tool to indicate the RV morphologic and functional improvement associated with better prognostic likelihood.

## Introduction

Pulmonary arterial hypertension (PAH) was a progressive disease that affected both pulmonary vasculature and heart. Although the initial damage in PAH may involve the pulmonary vasculature, the prognosis of patients with PAH is closely related to the right ventricular (RV) function (1–3). RV function is a great clinical determinant of the fate in patients with severe pulmonary hypertension (PH) (4, 5). The right heart (RH) failure may be a consequence of increased afterload in PH. An adapted right ventricle showed slightly dilated with preserved stroke volume and systolic function, whereas a maladapted right ventricle is dilated with reduced systolic function and increased dimensions (5, 6). Therefore, the changes of RV dimensions were inevitable and associated with pulmonary hemodynamics. Monitoring RV dimension could predict clinical worsening even at apparent clinical stability in PAH (7).

Echocardiography is an essential and non-invasive component estimated the role of RV function in PAH. Imaging modalities would be ideal to validate potential RV function and allow the creation of prediction scores to identify risk of mortality (8–10). Badagliacca et al. have reported the reversal of RH remodeling (RHRR) was associated with an improved outcome in idiopathic PAH (IPAH) patients by assessing right atrium (RA) area, left ventricular systolic eccentricity index (LV-EI), and RV end-diastolic area (11). Moreover, several clinical common echocardiographic variables were associated with mortality risk such as RV mid-diameter (RVMD) and tricuspid annular plane systolic excursion (12–14).

In the present study, we try to reassess and recalculate the efficacy of RH dimension's changes through general clinical echocardiographic parameters. Here, we defined a new model of attenuated RH remodeling (ARHR) using a decrease in RA area, RVMD, and left ventricular end-diastolic eccentricity index (LV-EId). Each of these echocardiographic parameters has been reported to be a determinant of prognosis in PAH (10, 11, 13). We proposed a hypothesis that ARHR created by a decrease in RA area, RVMD, and LV-EId would be associated with mortality and clinic outcomes.

## Materials and Methods

### Study Subjects and Design

Two hundred thirty-two consecutive treatment-naive adult IPAH patients (≥18 years of age at diagnosis) were enrolled and monitored at the time of their first right heart catheterization (RHC) in Shanghai Pulmonary Hospital from November 2010 to January 2018. IPAH was diagnosed according to guideline standard: a mean pulmonary artery pressure (mPAP) ≥25 mmHg and pulmonary vascular resistance (PVR) >3 Woods units at rest in the presence of a normal pulmonary artery wedge pressure (≤ 15 mmHg) on RHC (15, 16). In accordance with criteria, the respiratory function tests, perfusion lung scan, computed tomography scan, and echocardiography were used. If patients had definite causes of PAH, such as connective tissue disease and congenital heart disease, portopulmonary hypertension, chronic pulmonary thromboembolism, PH due to left heart diseases and lung diseases, and/or hypoxemia, they could be excluded.

**Figure 1:**
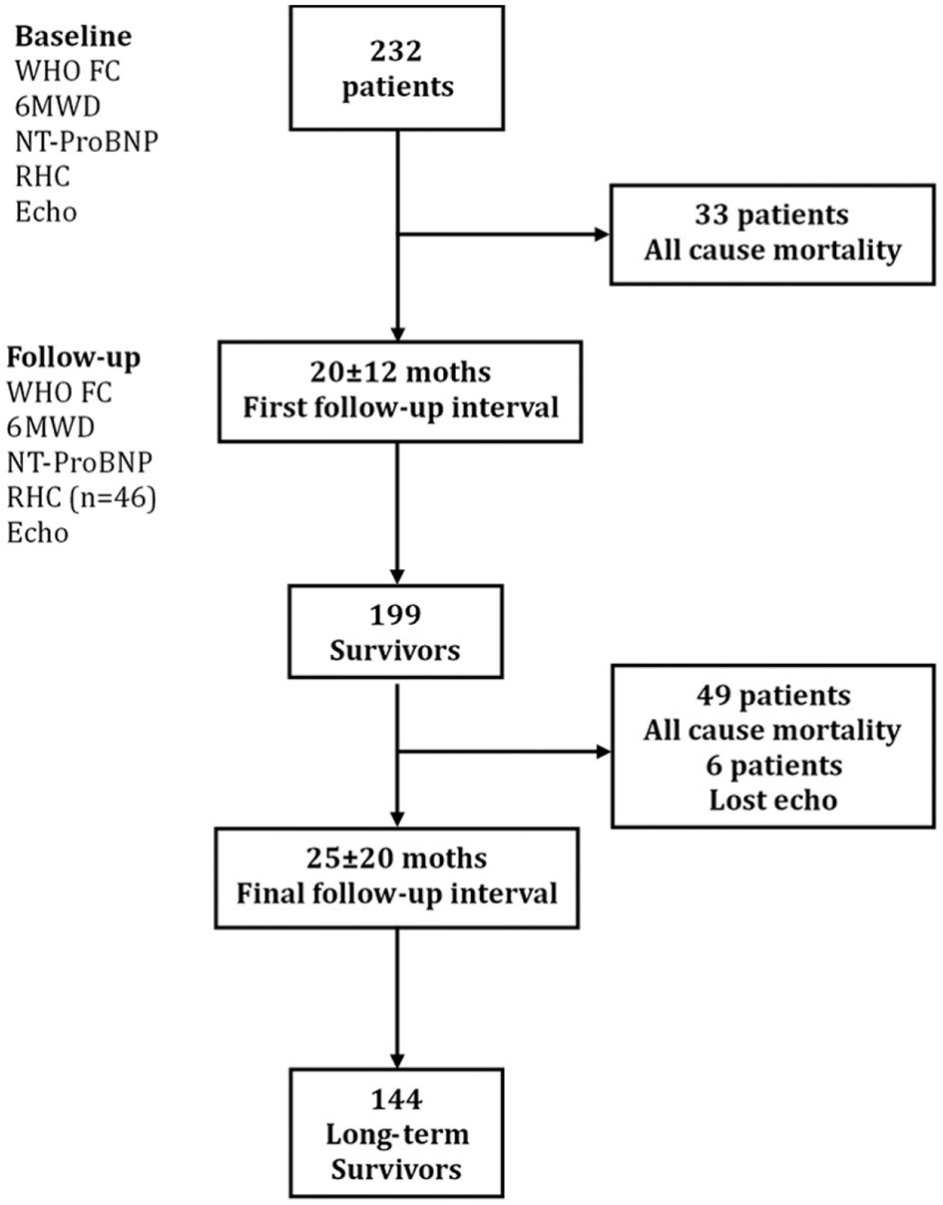
Study design flowchart. Echo, echocardiography; 6MWD, 6-min walking distance; NT-proBNP, N-terminal fragmental of pro–brain natriuretic peptide; RHC, right heart catheterization; WHO FC, World Health Organization functional class.

The baseline assessment at the time of diagnosis included medical history, physical examination, 6-min walking distance (6MWD), N-terminal fragmental of pro–brain natriuretic peptide (NT-proBNP), RHC, and echocardiography. During the first follow-up interval (mean follow-up time 20 ± 12 months), 33 patients died for all cause. The follow-up parameters included physical examination, 6MWD, NT-proBNP, echocardiography, and RHC (only 46 patients received RHC test). The 199 remaining survivors were reevaluated at a mean 25 ± 20 months until December 2018 (Figure 1). The major end point was all-cause mortality. The study was conformed according to the principles of the Declaration of Helsinki and was approved by the ethics committee of Shanghai Pulmonary Hospital (no. K16-293). Written informed consent signatures were obtained from all patients.

### RHC and Echocardiographic Assessment

Pulmonary hemodynamics were examined in triplicate at end-expiration using triple-lumen balloon-tipped thermodilution Swan–Ganz catheters. Cardiac output was detected by thermodilution (15, 16). Baseline echocardiographic measurements were performed within 24–48 h of the RHC. All echocardiographic data were acquired using commercially available equipment (Vivid 7, GE Healthcare) in standard views. The results were reviewed by at least three echocardiographic experts. Measurements were obtained from the mean of three consecutive beats based on the American Society of Echocardiography guidelines (17). The echo parameters and derived assessments that we focused on common and widely available for daily clinical practice, including RA area, RVMD, RV longitudinal diameter (RVLD), right atrial pressure (RAP), left atrium end-systolic diameter (LAESD), left ventricular end-diastolic diameter (LVEDD), left ventricular ejection fraction (LVEF), LV-EId, left ventricular end-systolic diameter (LVESD), pulmonary arterial systolic pressure (PASP), tricuspid annular plane systolic excursion (TAPSE), and presence of pericardial effusion. Spectral continuous-wave Doppler signal of tricuspid regurgitation corresponding to the RV-RA pressure gradient. SPAP was calculated as the sum of the estimated RAP and the peak pressure gradient between the peak RV and RA, as estimated by application of the modified Bernoulli equation to peak velocity represented by the tricuspid regurgitation Doppler signal. Early diastolic transmitral flow velocity (E) and late diastolic transmitral flow velocity (A) were measured by Doppler echocardiography. ARHR was defined by echocardiographic parameters of RA area, RVDM, and LV-EId, according to Cox proportional hazards regression for mortality risk at follow-up.

RVMD was defined as transversal RV diameter in the middle third of RV inflow, approximately hallway between the maximal basal diameter and the apex, at the level of papillary muscles at end-diastole (18). RA area is traced at the end of ventricular systole from the lateral aspect of the tricuspid annulus to the septal aspect, excluding the area between the leaflets and annulus, as well as the inferior vena cava, superior vena cava, and RA appendage (17). LV-EId was measured in the parasternal short-axis view at end-diastole. This index was calculated as D2/D1, where D2 is the minor-axis dimension of the left ventricle parallel to the septum, and D1 is the minor-axis dimension perpendicular to and bisecting the septum (3). TAPSE is measured by M-mode echocardiography with the cursor optimally aligned along the direction the tricuspid lateral annulus in the apical four-chamber view (18).

### Statistical Analysis

Continuous variables were expressed as means with corresponding standard deviations, and categorical variables were expressed as numbers and percentages. The proportions were compared with the χ^2^ test. If the data were normally distributed, two-group comparisons were performed with unpaired or paired, two-tailed *t*-test for means. If the data were not normally distributed, non-parametric two-sided Mann–Whitney *U* test was used. Bivariate linear analysis was to evaluate the correction between the change of NT-proBNP, 6MWD, and RA area, RVMD, and LV-EId during the follow-up, fitting curve was used a quadratic model with mean value 95% confidence interval.

Cox proportional hazards regression was used to determine risk factors for mortality at follow-up and to identify the association among patient characters and outcomes. For optimal cutoff value for mortality, RA area, RVMD, and LV-EId were generated by receiver operating characteristic (ROC) curves. The Cox proportional hazards regression used to derive a risk calculator assigning weighted for three echo parameters. An integer score of RA area was assigned a value of 1 for the β-coefficient associated with a hazard ratio (HR) of 1.009. Integer scores of 1.621 for RVMD and 2.033 for LV-EId were created assigning values of 1.5 and 2.0, respectively. The total sum of three echo parameters was used for each patient based on the number of the echo cutoff value. Univariate and multivariate logistic regression analyses were chosen to identify clinical and hemodynamic determinants of ARHR. Multivariate analysis to WHO FC I–II, 6MWD, and NT-proBNP for model 1 was created, and WHO FC I–II plus ARHR for model 2. The C-statistic was calculated for each model and model discrimination by R version 2.11.1 (19).

The French non-invasive low-risk criterion was calculated based on the number of non-invasive criteria to derive the original model 1, including WHO FC I–II, 6MWD >440 m, NT-proBNP <300 ng/L (20). The French non-invasive low-risk criteria score was used for Cox regression analysis to predict mortality (model 1), and model 2 was added the echo score. Survival analyses were performed using the Kaplan–Meier method and were compared by means of the log-rank test. For all analyses, *p* < 0.05 was considered statistically significant. All calculations were performed using the SPSS 14.0 statistical software package (Statistical Package for the Social Sciences, Chicago, IL, USA).

## Results

### Baseline Clinical and Hemodynamic Characteristics of Patients

The baseline clinical, hemodynamic, and echocardiographic features of the IPAH patients are summarized in Table 1. Among 232 patients with IPAH, 147 (71%) were women, and 153 (66%) in WHO FC III and IV, with impaired exercise capacity and severe PH hemodynamic status. The echocardiography examination at baseline presented severe RV dilatation and systolic function reduction. Most patients had mild to moderate tricuspid regurgitation.

**Table 1:** Baseline clinical, hemodynamic, and echocardiographic characteristics of patients with IPAH.

**Variable**	**Mean ± SD or no. (%) (*n* = 232)**
Age, years	40 ± 15
Female, *n* (%)	147 (71)
BMI, kg/m^2^	22 ± 4.7
WHO FC, *n* (%)	
Class I–II	79 (34)
Class III	142 (61)
Class IV	11 (5)
6MWD, m	390 ± 107
NT-proBNP, ng/L	997± 1,088
Hemodynamics	
RAP, mmHg	7 ± 4.9
mPAP, mmHg	59 ± 15
PAWP, mmHg	8 ± 3.1
CI, L/min per m^2^	2.6 ± 0.8
PVR, Woods units	14 ± 6.5
S_V_O_2_, %	62 ± 9.1
Echocardiography	
RA area, cm^2^	25 ± 11
RVMD, cm	4.5 ± 0.8
LV-EId	1.6 ± 0.4
RVLD, cm	6.5 ± 0.9
RA major axis dimension, cm	5.3 ± 1.0
RA minor axis dimension, cm	4.9 ± 1.2
LVESD, cm	2.2 ± 0.5
LVEDD, cm	3.8 ± 0.6
LAESD, cm	3.1 ± 0.5
TAPSE, mm	17 ± 3.4
LV-E wave PW, cm/s	54.9 ± 18.7
LV-A wave PW, cm/s	58.9 ± 17.1
LVEF, %	74 ± 8.4
PASP, mmHg	86 ± 23
RAP, mmHg	7 ± 3
Pericardial effusion, *n* (%)	63 (27)
Initial specific therapies, *n* (%)	
No specific/CCB therapy	16 (7)
Monotherapy	145 (63)
ERA	35 (15)
PDE5i	98 (42)
Prostanoid	12 (5)
Dual combination	71 (31)

*Values are expressed as medians (interquartile range) or n (%), unless otherwise stated*.

*BMI, body mass index; CCB, calcium channel blocker; CI, cardiac index; ERA, endothelin receptor antagonist; IPAH, idiopathic pulmonary arterial hypertension; LAESD, left atrium end-systolic diameter; LV A wave PW, pulsed wave left ventricular A wave; LVEDD, left ventricular end-diastolic diameter; LVEF, left ventricular ejection fraction; LV-EId, left ventricular end-diastolic eccentricity index; LVESD, left ventricular end-systolic diameter; LV E wave PW, pulsed wave left ventricular E wave; mPAP, mean pulmonary arterial pressure; 6MWD, 6-min walking distance; NT-proBNP, N-terminal fragmental of pro–brain natriuretic peptide; PAWP, pulmonary artery wedge pressure; PASP, pulmonary arterial systolic pressure; PDE5i, phosphodiesterase type 5 inhibitor; PVR, pulmonary vascular resistance; RA area, right atrium area; RAP, right atrial pressure; RVLD, right ventricular longitudinal diameter; RVMD, right ventricular mid diameter; TAPSE, tricuspid anular plane systolic excursion; SvO_2_, mixed venous oxygen saturation; WHO FC, World Health Organization functional class*.

During mean 20 ± 12 follow-up interval, 33 patients (14%) died, including 26 deaths directly related to RH failure, 5 sudden deaths, and 2 cases not able to be ascertained. Compared with the remaining 199 patients, these patient deaths at baseline were more severe and had advanced disease, such as PVR (16 ± 10 vs. 13 ± 6 Woods unit, *p* = 0.01), RAP (9 ± 6 vs. 6 ± 5 mmHg, *p* = 0.03), S_V_O_2_ (60 ± 9 vs. 64 ± 9 %, *p* = 0.02), mPAP (62 ± 17 vs. 58 ± 15 mmHg, *p* = 0.24), CI (2.4 ± 0.8 vs. 2.7 ± 0.8 L/min per m^2^, *p* = 0.16), NT-proBNP (1,341 ± 974 vs. 964 ± 1,092 ng/L, *p* = 0.03), WHO FC (3.0 ± 0.6 vs. 2.6 ± 0.6, *p* = 0.04), and 6MWD (356 ± 106 vs. 394 ± 110 m, *p* = 0.09).

Clinical and echocardiographic information was available for 199 survivors at the mean first follow-up interval.

### Clinical and Echocardiographic Findings at First Follow-Up

At the first follow-up reevaluation, the 199 surviving patients had a relative improvement in clinical condition (6MWD, +37 ± 71 m, *p* = 0.02; NT-proBNP, −361 ± 652 ng/L, *p* = 0.025; WHO FC, −0.2 ± 0.1, *p* = 0.53) and hemodynamics (PVR, −3.3 ± 1.9 Woods unit, *p* = 0.004; mPAP, −10.6 ± 9.3 mmHg, *p* = 0.03; CI, +0.5 ± 0.2 L/min per m^2^, *p* = 0.005; RAP, −2.0 ± 1.5 mmHg, *p* = 0.39; S_V_O_2_, +5.9 ± 4.0 %, *p* = 0.13; follow-up RHC samples were from 46 patients). Importantly, these patients had a significant improvement of most echocardiographic parameters (RA area, −4.2 ± 3.8 cm^2^, *p* = 0.010; RVMD, −0.3 ± 0.1 cm, *p* = 0.015; LV-EId, 0.09 ± 0.04, *p* = 0.03; TAPSE, +0.24 ± 0.49, *p* = 0.027; RA major axis dimension, 0.17 ± 0.37 cm, *p* = 0.001; RA minor axis dimension, 0.19 ± 0.33 cm, *p* = 0.011; LVEF, 6.0 ± 3.0 %, *p* < 0.001; LV-E wave, 8.0 ± 3.4 cm/s, *p* = 0.001; LV-E wave, 2.2 ± 4.5 cm/s, *p* = 0.51; pericardial effusion 8% regression, *p* = 0.002) compared with their baseline data.

### ARHR and Determinants

At univariate analysis, absolute changes from baseline to the first follow-up assessment in RA area (HR, 1.009; 95% confidence interval, 0.991–1.027; *p* = 0.01), RVMD (HR, 1.621; 95% confidence interval, 1.083–2.427; *p* = 0.01), and LV-EId (HR, 2.033; 95% confidence interval, 0.386–3.524; *p* = 0.02) were predictive of all-cause death in the subsequent period. The optimal cutoff points by ROC analysis protective against all-cause death were −5.8 cm^2^ (sensitivity, 75%; specificity, 66%) for RA area change, −0.7 cm (sensitivity, 77%; specificity, 68%) for RVMD change, and −0.4 (sensitivity, 86%; specificity, 67%) for LV-EIs change.

**Figure 2:**
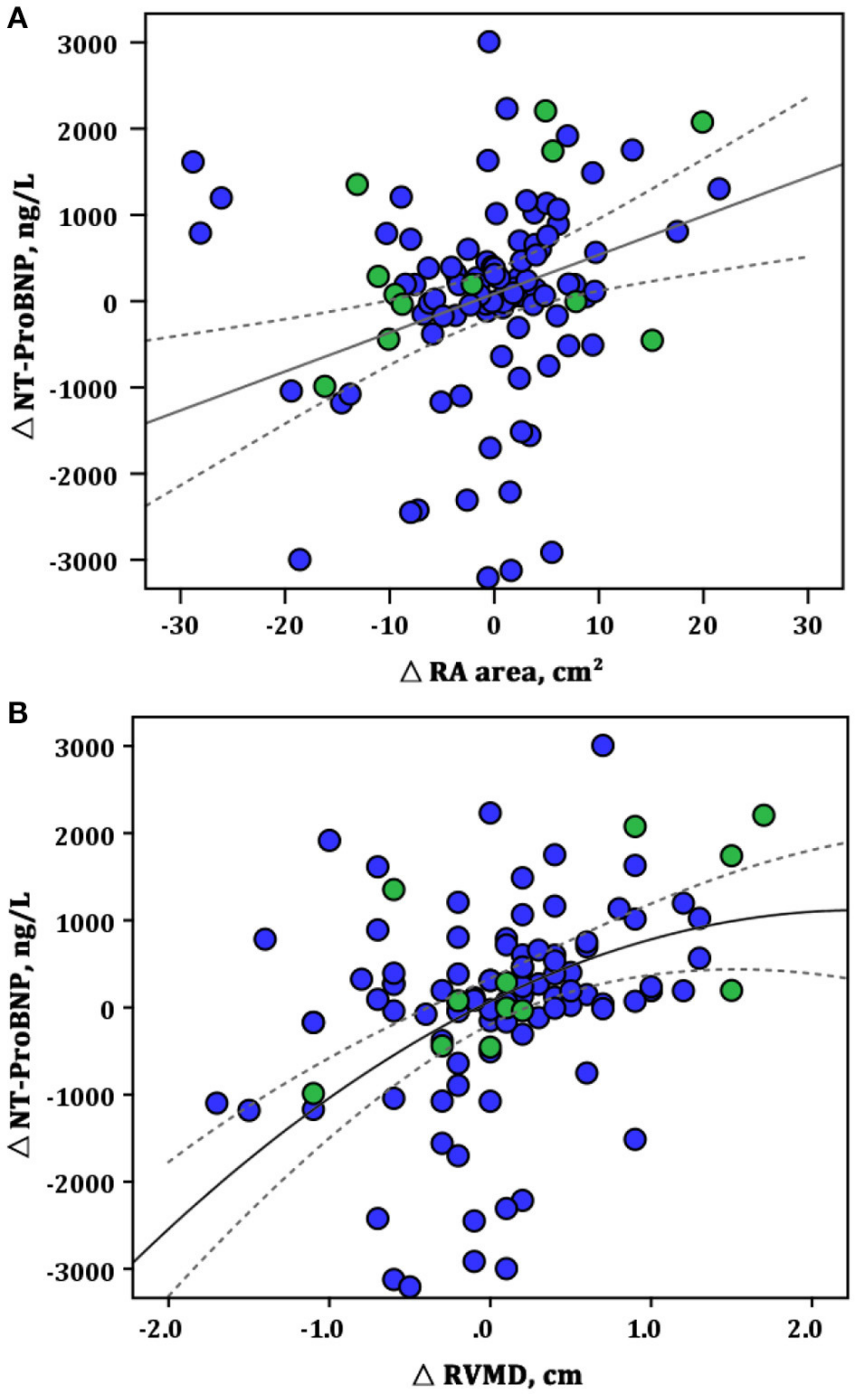
Correlations between the changes in RA area, RVMD, and NT-proBNP at the first follow-up assessment. **(A)** ΔRA area vs. Δ NT-proBNP (linear model: *r*^2^ = 0.51, *p* = 0.009); **(B)** ΔRVMD vs. Δ NT-proBNP (linear model: *r*^2^ = 0.45, *p* = 0.001). Green circles represent the patients with ARHR; blue circles, without ARHR. NT-proBNP, N-terminal fragmental of pro–brain natriuretic peptide; RA, right area; RVMD, right ventricular mid diameter.

**Figure 3:**
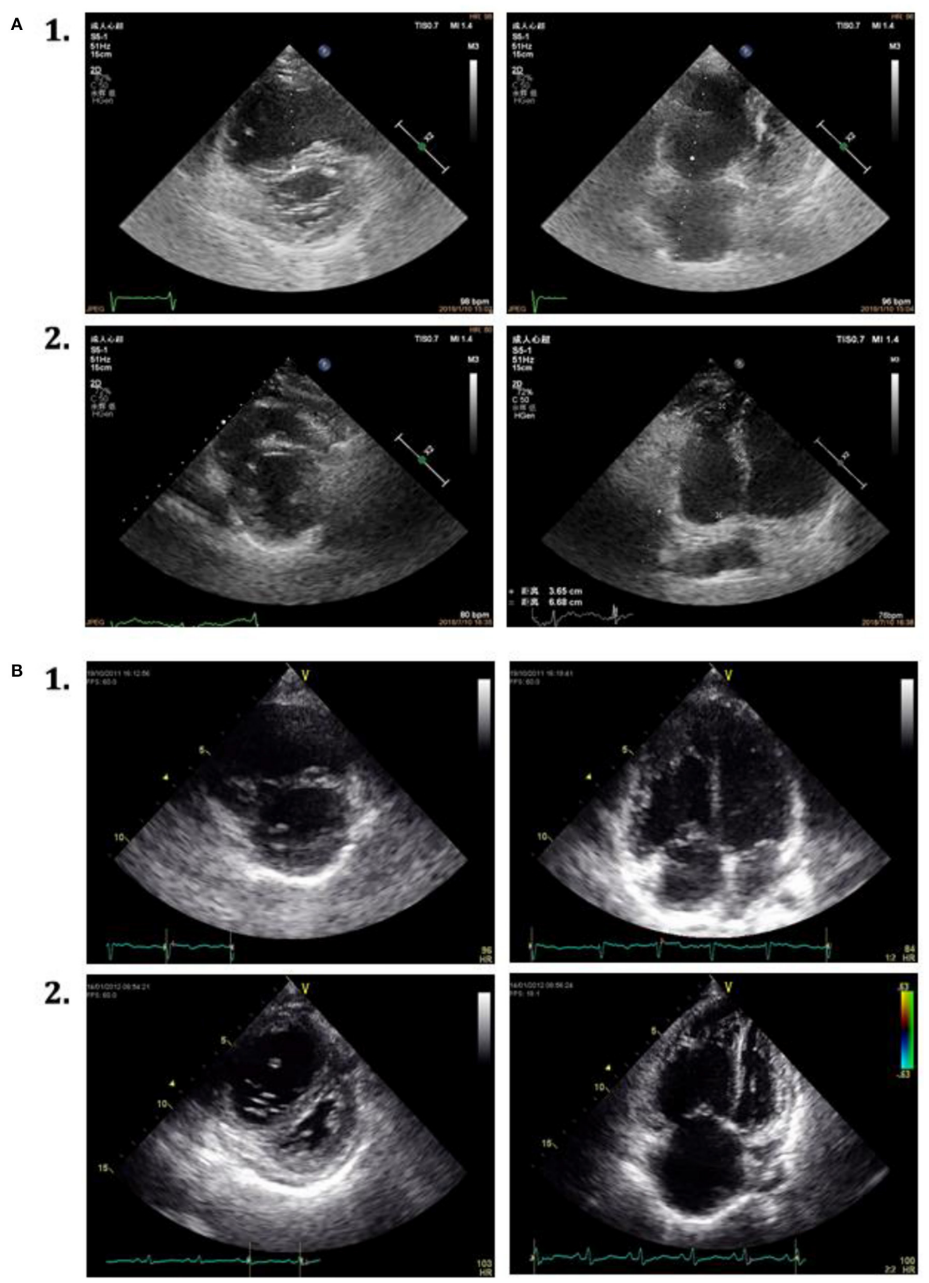
Echocardiographic parasternal short-axis view and apical four-chamber view in 2 patients with IPAH. **(A)** 1. The characteristics at baseline. 2. Attenuated right heart remodeling of the same patient at the first follow-up assessment. **(B)** 1. The characters at baseline. 2. Without attenuated right heart remodeling of the same patient at the first follow-up assessment.

A score was created deriving integers according to the HRs of the latter echo variables. Based on the achievement of change cutoff points of echo parameters, patients are categorized by the echo score. One hundred thirty-four patients (67.3%) had a score between 0 and 2.0 (0 or 1 protective changes cutoff point of echo parameters), 30 (15.1%) had a score between 2.5 and 3.5 (achievement of 2-echo-parameters cutoff point), and 35 (17.6%) had a score between 4.0 and 4.5 (achievement of all 3-echo-parameters cutoff point). The score between 4.0 and 4.5 was selected as a comprehensive criterion for ARHR. Conversely, a score <4.0 was defined as without ARHR. There were no significant differences in clinical and echocardiographic parameters between patients with or without subsequent ARHR at the first follow-up interval (Table 2). At the first follow-up, a significant correlation was present between the change of NT-proBNP and improvement of RA area (*r*^2^ = 0.51, *p* = 0.009) and RVMD (*r*^2^ = 0.45, *p* = 0.001) (Figure 2). Two examples of patients with and without ARHR at the first follow-up are demonstrated at Figure 3.

**Table 2:** Clinical, hemodynamic, and echocardiographic characteristics of two patient groups based on ameliorative right heart remodeling at first follow-up interval.

**Variable**	**No ARHR (*n* = 164)**	**ARHR (*n* = 35)**	***p*-value**
Age, years	42 ± 17	37 ± 11	0.16
Female, *n* (%)	131 (75)	18 (75)	0.89
WHO FC, *n* (%)			0.22
Class I–II	56 (32)	9 (38)	
Class III	108 (62)	14 (58)	
Class IV	11 (6)	1 (4)	
6MWD, m	415 ± 115	389 ± 131	0.92
NT-proBNP, ng/L	805 ± 1,141	1,023 ± 1,297	0.74
Echocardiography			
RA area, cm^2^	28 ± 12	23 ± 13	0.06
RVMD, cm	5.1 ± 0.9	4.5 ± 0.8	0.28
LV-EId	1.6 ± 0.5	1.5 ± 0.3	0.24
RVLD, cm	6.6 ± 0.8	6.5 ±0.7	0.59
RA major axis dimension, cm	5.5 ± 1.4	6.2 ± 1.3	0.72
RA minor axis dimension, cm	5.0 ± 1.4	5.3 ± 1.4	0.79
LVESD, cm	2.0 ± 0.6	1.5 ± 0.4	0.14
LVEDD, cm	3.7 ± 0.7	3.2 ±0.2	0.13
LAESD, cm	3.1 ± 0.5	3.0 ± 0.3	0.34
TAPSE, mm	17 ±4.2	13 ± 6.6	0.46
LV-E wave PW, cm/s	61.6 ± 18.1	58.2 ± 21.1	0.44
LV-A wave PW, cm/s	61.7 ± 19.4	71.7 ± 10.7	0.86
LVEF, %	78 ± 9.6	81 ± 6.7	0.40
PASP, mmHg	83 ± 24	90 ± 12	0.49
RAP, mmHg	7 ±3	8 ± 5	0.21
Pericardial effusion, *n* (%)	53 (30)	13 (54)	0.22
Initial specific therapies, *n* (%)			
No specific/CCB therapy	6 (3)	1 (4)	
Monotherapy			
ERA	25 (14)	4 (17)	
PDE5i	47 (27)	5 (21)	
Prostanoid	7 (4)	1 (4)	
Dual combination	88 (50)	13 (54)	

*Values are expressed as medians (interquartile range) or n (%), unless otherwise stated*.

*ARHR, attenuated right heart remodeling; CCB, calcium channel blocker; ERA, endothelin receptor antagonist; LAESD, left atrium end-systolic diameter; LV A wave PW, pulsed wave left ventricular A wave; LVEDD, left ventricular end-diastolic diameter; LVEF, left ventricular ejection fraction; LV-EId, left ventricular end-diastolic eccentricity index; LVESD, left ventricular end-systolic diameter; LV E wave PW, pulsed wave left ventricular E wave; 6MWD, 6-min walking distance; NT-proBNP, N-terminal fragmental of pro–brain natriuretic peptide; PASP, pulmonary arterial systolic pressure; PDE5i, phosphodiesterase type 5 inhibitor; RA area, right atrium area; RVLD, right ventricular longitudinal diameter; RVMD, right ventricular mid diameter; TAPSE, tricuspid anular plane systolic excursion; WHO FC, World Health Organization functional class*.

### ARHR and Prognosis

After the first follow-up evaluation, the remaining 199 surviving patients were monitored for a mean of 25 ± 20 months. During this period, there were 55 patient deaths. The total survival rate at the final follow-up assessment was 85, 70, and 53% at 1, 3, and 5 years of follow-up, respectively.

**Figure 4:**
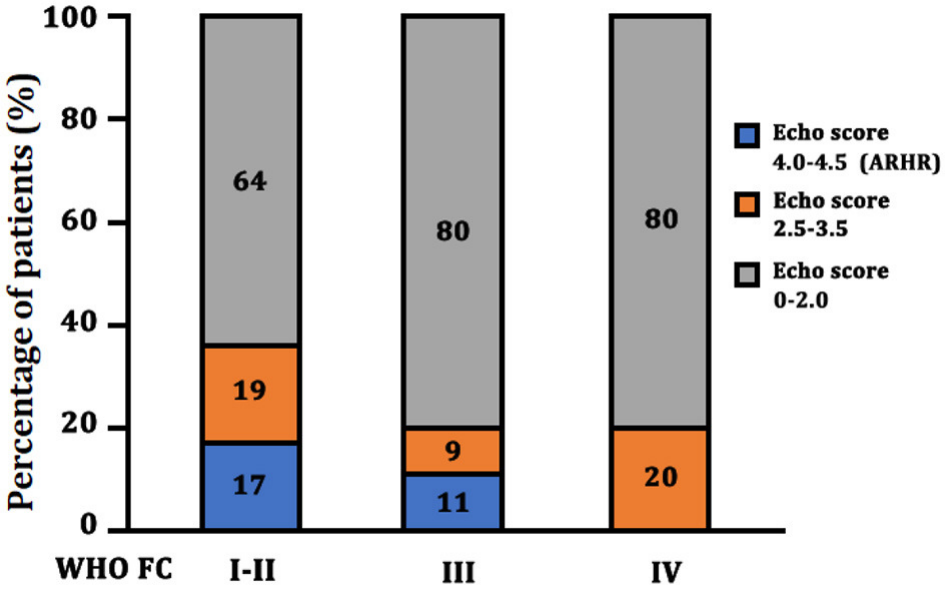
Echo score in different WHO FC group at the follow-up assessment. ARHR, attenuated right heart remodeling; Echo, echocardiography; WHO FC, World Health Organization functional class.

As shown in Table 3, we generated two Cox regression models at the follow-up assessment. Model 1 demonstrated that WHO FC I and II and NT-proBNP were independent predictors of death. Model 2 was created by adding the echo score according to the 3 echo parameters, showing the ARHR and WHO I and II were significantly protective factors independently from other variables. Accordingly, there were a greater proportion of patients attaining ARHR in WHO FC I–II group and lesser proportion of ARHR patients in WHO FC III (*p* = 0.01) (Figure 4). No ARHR patients were in the WHO FC IV group.

**Table 3:** Cox regression models for dead prediction at the first follow-up evaluation: model 1 and model 2.

**Variable**	**Unit**	**HR (95% confidence interval)**	***p-*value**	**C-statistic (95% confidence interval)**
Model 1				0.60 (0.52–0.73)
WHO I–II		0.46 (0.21–0.97)	0.0001	
6MWD	1	0.99 (0.98–1.02)	0.07	
NT-proBNP	1	1.46 (1.27–3.14)	0.002	
Model 2				0.75 (0.69–0.82)
WHO I–II		0.55 (0.21–0.98)	0.0001	
Echo score^a^				
0-2	REF			
2.5–3.5		0.80 (0.39–1.96)	0.45	
4–4.5 (ARHR)		0.42 (0.21–0.88)	0.004	

*ARHR, attenuated right heart remodeling; CI, confidence interval; HR, hazard ratio; 6MWD, 6-min walking distance; NT-proBNP, N-terminal fragmental of pro–brain natriuretic peptide; WHO FC, World Health Organization functional class*.

a *Echo score, score based on protective changes in echo parameters by ROC curve analysis cutoff value*.

**Figure 5:**
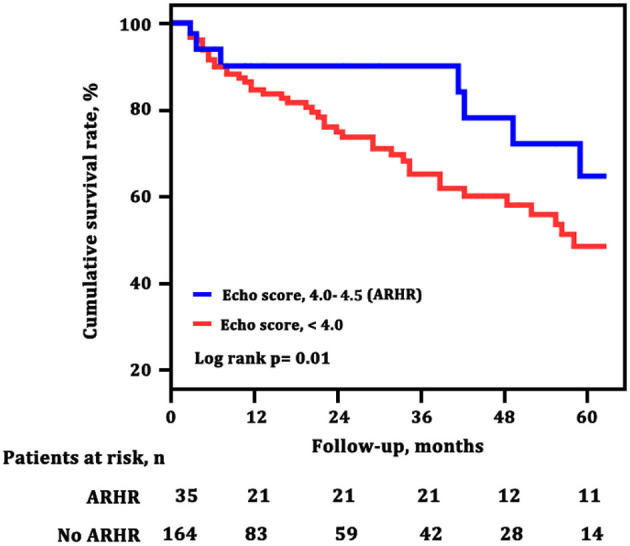
Survival estimates of patients with and without attenuated right heart remodeling (ARHR). Green line (echo score, 4.0–4,5) represents the survival of patients with ARHR; blue line (echo score, <4.0) represents the survival of patients without ARHR.

The survival curves at final follow-up of 199 surviving patients classified according to ARHR are shown in Figure 5. Patients with ARHR had a better long-term survival than others (log-rank *p* = 0.01). The cumulative survival rates at 1, 3, and 5 years of follow-up were 89, 89, and 68% in patients with ARHR, respectively, and 84, 65, and 41% in patients without ARHR.

### ARHR Combined With French Non-invasive Low-Risk Criteria

**Figure 6:**
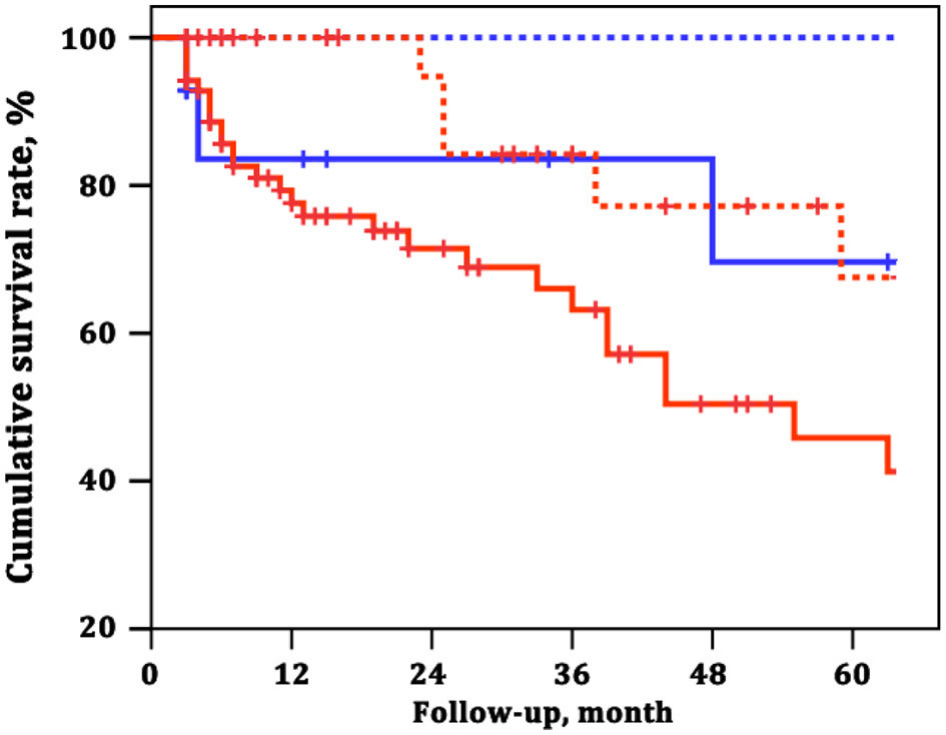
Survival estimates of the four groups of patients based on the combination of French non-invasive low-risk criteria and attenuated right heart remodeling (ARHR). Blue dashed line represents French non-invasive criterion 3 and echo score 4.0–4.5. Red solid line represents French non-invasive criteria 0–2 and echo score 4.0–4.5. Blue solid line represents French non-invasive criterion 3 and echo score <4.0. Red dashed line represents French non-invasive criteria 0–2 and echo score <4.0.

To explore the adding value of ARHR on a well-generated risk evaluation tool, we repeated the analysis building a first model 1 according to the number of French non-invasive low-risk criteria (WHO FC I–II; 6MWD >440 m; NT-proBNP <300 ng/L). The ARHR echo score was then added to model 2 was and showed prognostic strength power (Table 4). The survival of the four groups are shown in Figure 6, based on the combination of French non-invasive low-risk criteria (3 criteria vs. 0–2 criteria) and ARHR (echo score 4.0–4.5 vs. <4.0). Patients with ARHR and French non-invasive criterion 0 had the best prognosis; 1-, 3-, and 5-year survival rates were all 100%. Patients without ARHR (score <4) and French non-invasive criteria 0–2 presented worst survival, and 1-, 3-, and 5-year survival rates were 78, 63, and 46%, respectively. However, we did not find significant difference between the combination of ARHR (score 4.0–4.5) and French non-invasive criterion 0–2 and those of non-ARHR (score <4.0) and French non-invasive criteria 3 (Figure 6).

**Table 4:** Cox regression models for dead prediction according to the French non-invasive risk assessment and echo.

**Variable**	**Unit**	**HR (95% confidence interval)**	***p*-value**	**C-statistic (95% confidence interval)**
Model 1				0.61 (0.53–0.73)
French non-invasive low-risk criteria^a^				
3 criteria	REF			
1–2 criteria		3.03 (1.10–4.28)	0.031	
0 criteria		2.77 (0.84–3.15)	0.041	
Model 2				0.72 (0.67–0.80)
French non-invasive low-risk criteria				
3 criteria	REF			
1–2 criteria		2.77 (1.06–3.04)	0.092	
0 criteria		2.97 (0.89–3.85)	0.042	
Echo score^b^				
0–2	REF			
2.5–3.5		0.83 (0.22–1.79)	0.655	
4–4.5 (ARHR)		0.39 (0.12–0.77)	0.012	

a *French non-invasive low-risk criteria: based on the number of non-invasive criteria (WHO FC I–II; 6MWD >440 m; NT-proBNP <300 ng/L)*.

b *Echo score: score based on protective changes in echo parameters by ROC curve analysis cutoff value*.

## Discussion

Echocardiographic RV imaging combined with pulmonary hemodynamics was a good framework to interpret the prognosis of patients with IPAH (1, 4). Monitoring the change of RV dimensions back to normal or improvement should be useful for evaluation of the RV function. Therefore, it is noteworthy to find a practical echocardiographic predictor tool to remind prognosis. In our study, we defined an ARHR model and found that at the first reevaluation (a) ARHR was an independent predictor of mortality; and (b) ARHR combined with French non-invasive criterion could better predict the outcome of death. The RHRR might serve as a tool for pending prognosis in patients with IPAH.

RA area, RVMD, and LV-EId selected in this study were conventional and important echocardiography indices (12, 17, 21–24). For example, RA area >18 cm^2^ was considered as one of the preferred parameters for end-diastole RA enlargement (17). RA enlargement reflected the severity of RH failure and predicted adverse outcomes in patients with severe primary PH (3). A study from Badagliacca's team used RA area as one of the determinants of RV reverse remodeling (10). Therefore, our findings supposed the change of RA area was also a marker of RA dilatation. If there is RV dilatation, RVMD should be measured to respond the chronic volume and/or pressure overload (22). In our study, RV size was measured from a four-chamber view, where RVMD was easily obtained and markers of RV dilatation. The third important parameter in this study is LV-EId, reflecting the degree of septal shift in diastole (3, 10). Echocardiography showed improved LV-EId in proportion to treatment-induced decrease in PVR (24). Taken together, the shift in RV remodeling during the development of PAH is not well elucidated. It is challenging to determine the best parameters for reflecting RV failure progression (25). ARHR in this study might be an indication with reversal of RH dimensions.

It is recognized that the change of RV structure is the main predictor of poor clinical outcomes in PAH (22). There was no more than 18% of IPAH patients presented with ARHR after a mean of 20 ± 12 months in our study, despite that more than 93% of patients had received PAH-specific therapies. This result is similar with the study on RHRR in IPAH patients after 1-year targeted treatment, which implies that the reversal of RV remodeling is hard and complex (10). At first follow-up time, both the disease severity and echocardiographic indicators seemed to have no significant difference between ARHR and no-ARHR group. However, the patients with ARHR had better long-term survival, as longstanding increase of RV afterload will overwhelm the compensatory mechanisms of the RV (26). Not surprisingly, the patients' hemodynamic status of pulmonary circulation is not always consistent with the changes of RV structure and function. Despite hemodynamics deterioration in patients with PAH, RV contractility is usually increased and not decreased (4, 27, 28). Consequently, the amount of work for the RV remained unaltered, leading to a clinical improvement but unchanged prognosis (29). Therefore, non-invasive imaging of RV dimensions and function is important to the longitudinal monitoring of patients with PAH and continued understanding of the response of RV to pulmonary vascular remodeling (30).

French PH registry permitted to use three non-invasive variables to assess the low-risk criteria score, such as WHO FC, 6MWD, and BNP/NT-proBNP (20). However, it remained unclear whether the addition of other non-invasive modes, such as echocardiography, to the three non-invasive criteria could further improve the prognostic utility (20, 31, 32). Notably, in our study, echocardiography-determined ARHR was able to further stratify patients assessed with French non-invasive low-risk criteria score, suggesting a better prognosis for those patients achieving ARHR. The 1-, 3-, and 5-year survival rates were all 100% in patients with ARHR and French non-invasive criterion 0, compared with 78%, 63%, and 46% in patients with no ARHR (score <4) and French non-invasive criteria 1–4, respectively. Therefore, our results indicated that non-invasive French low-risk criteria combined with echocardiography ARHR would be a preferable predictor model for mortality in patients with IPAH.

Certainly, several echocardiographic parameters were related to long-term prognosis, such as TAPSE, PASP, etc. (12, 33, 34). However, changes in TAPSE or PASP were not predictive of mortality at univariate analysis in our first follow-up time. This is attributable to the limitation of TAPSE assuming that the displacement of a single segment represents the function of a complex 3D, considering the RV shape is more “regular” (35, 36). Indeed, patients in our study underwent more pronounced increases in RV afterload (severe RV dilation), especially for non-ARHR patients who did not have significant improvement for systolic function. Thus, echocardiography is still a comprehensive and multiple tool for non-invasive assessment of the RH.

## Study Limitations

There are several limitations to this study. First, this is a retrospectively study in a single center, and the sample size is not large enough with a potential selection bias. The follow-up intervals of PAH patients were not fixed and varied. Second, the follow-up intervals of patients are not standardized and lack of RHC hemodynamic testing. It is different to further analyze the relationship between the change of hemodynamic parameters and ARHR. Then, we did not select the best ROC curve cutoff values for subsequent analysis to avoid the potential risk of a type I error. Finally, there are limitations to the quantification of RH morphology and function using two-dimensional echocardiography. In the future, we need more and accurate parameters to evaluate RV function.

## Conclusions

In summary, our study demonstrated that echocardiographic ARHR created by RA area, RVMD, and LV-EId was an independent predictor of long-term prognosis in patients with IPAH. Similarly, ARHR integrated with French non-invasive criterion could better predict the risk for mortality. ARHR might be a useful tool to indicate RV morphologic and functional improvement associated with better prognostic likelihood. Whether this increases the proportion of patients with ARHR remained to be further confirmed in prospective and multicenter assessments.

## Data Availability Statement

The original contributions presented in the study are included in the article/supplementary material, further inquiries can be directed to the corresponding authors.

## Ethics Statement

The studies involving human participants were reviewed and approved by Ethic Committee of Shanghai Pulmonary Hospital. The patients/participants provided their written informed consent to participate in this study. Written informed consent was obtained from the individual(s) for the publication of any potentially identifiable images or data included in this article.

## Author Contributions

RZ and LW were directly involved in the patients' recruitment and care, contributed to the study design, study conduct and supervision, scientific overview, data analysis, and editing of the manuscript. Q-HZ, S-GG, and RJ contributed to patient enrolment, data analysis, scientific interpretation, drafting, and editing the original manuscript. CL, G-FC, C-JL, H-LQ, and J-ML contributed to recruitment of participants, data collection and curation, and formal analysis. All authors have reviewed the manuscript and approved the final version for submission.

## Conflict of Interest

The authors declare that the research was conducted in the absence of any commercial or financial relationships that could be construed as a potential conflict of interest.

## Abbreviations

BMI: body mass index
CI: cardiac index
IPAH: idiopathic pulmonary arterial hypertension
LAESD: left atrium end-systolic diameter
LV A wave PW: pulsed wave left ventricular A wave
LVEDD: left ventricular end-diastolic diameter
LVEF: left ventricular ejection fraction
LV-EId: left ventricular end-diastolic eccentricity index
LVESD: left ventricular end-systolic diameter
LV E wave PW: pulsed wave left ventricular E wave
mPAP: mean pulmonary arterial pressure
6MWD: 6-min walking distance
NT-proBNP: N-terminal fragmental of pro–brain natriuretic peptide
PAWP: pulmonary artery wedge pressure
PASP: pulmonary arterial systolic pressure
PVR: pulmonary vascular resistance
RA area: right atrium area
RAP: right atrial pressure
RVLD: right ventricular longitudinal diameter
RVMD: right ventricular mid diameter
TAPSE: tricuspid annular plane systolic excursion
SvO_2_: mixed venous oxygen saturation
WHO FC: World Health Organization functional class.
